# Characteristics of Type 1 Diabetes Among Patients Carrying the Protective HLA‐DQB1*06:02 Allele

**DOI:** 10.1111/tan.15720

**Published:** 2024-11-20

**Authors:** Antti‐Mathias Taka, Taina Härkönen, Paula Vähäsalo, Tommi Vatanen, Johanna Lempainen, Riitta Veijola, Maaret Turtinen, Jorma Ilonen, Mikael Knip, the Finnish Pediatric Diabetes Register

**Affiliations:** ^1^ Pediatric Research Center, New Children's Hospital Helsinki University Hospital Helsinki Finland; ^2^ Research Program for Clinical and Molecular Metabolism, Faculty of Medicine University of Helsinki Helsinki Finland; ^3^ Research Unit of Clinical Medicine University of Oulu Oulu Finland; ^4^ Medical Research Center Oulu University Hospital and University of Oulu Oulu Finland; ^5^ Broad Institute of MIT and Harvard Cambridge Massachusetts USA; ^6^ Liggins Institute University of Auckland Auckland New Zealand; ^7^ Immunogenetics Laboratory, Institute of Biomedicine University of Turku Turku Finland; ^8^ Department of Pediatrics University of Turku and Turku University Hospital Turku Finland; ^9^ Clinical Microbiology Turku University Hospital Turku Finland; ^10^ Tampere Center for Child Health Research Tampere University Hospital Tampere Finland

**Keywords:** autoantibodies, children, genetic risk, HLA genotype, type 1 diabetes

## Abstract

We set out to examine in an observational study characteristics of type 1 diabetes at the time of diagnosis among paediatric patients carrying the protective HLA class II DQB1*06:02 allele. We compared characteristics of type 1 diabetes among 5530 Finnish children aged 0–14 years diagnosed between 2003 and 2018. Seventy‐five children with type 1 diabetes carried the DQB1*06:02 allele. The carriers of DQB1*06:02 allele were compared to all children with type 1 diabetes without this allele and those with a high‐risk genotype. We also analysed, how does the genotype of a high‐risk haplotype paired with DQB1*06:02 affect the phenotype of patients with newly diagnosed type 1 diabetes. Carriers of the DQB1*06:02 allele were diagnosed at an older age than those with any other HLA class II genotype (*p* = 0.003) or the high‐risk genotype (*p* < 0.001). After adjusting the results for age and sex, no significant differences in clinical markers were observed. Glutamic acid decarboxylase autoantibody (GADA) levels were higher among carriers of DQB1*06:02 when compared to those with other genotypes (*p* = 0.033). Having a high‐risk haplotype paired with DQB1*06:02‐positive haplotype was associated with higher levels of islet antigen 2 autoantibodies (IA‐2A) (*p* < 0.001) and somewhat shorter duration of symptoms (*p* = 0.043). The association between the protective DQB1*06:02 allele and an older age at diagnosis as well as higher levels of GADA at diagnosis of type 1 diabetes was confirmed. The effects of the DQB1*06:02‐positive haplotype seem to dominate when paired with a high‐risk haplotype.

## 1 Introduction

Type 1 diabetes (T1D) is an autoimmune disease the incidence rate of which varies greatly depending on the population studied. Finland has the highest incidence in the world [1]. The current conception is that individuals carry a variable predisposition to T1D based on their genetics. For progression to overt disease later in life, an external environmental factor is needed as a trigger. The genetic background of T1D is well established, with the HLA class II DR/DQ region being the most notable element accounting for approximately half of the genetic risk [2, 3]. In addition, more than 70 single nucleotide polymorphisms have been reported to be associated with susceptibility to T1D [4]. Certain haplotypes, such as DRB1*04:01/02/04/05‐DQA1*03‐DQB1*03:02 and DRB1*03:01‐DQA1*05‐DQB1*02, have been shown to inflict high risk for T1D, whereas some haplotypes offer protection from the disease. DRB1*15‐DQA1*01‐DQB1*06:02 (*DR15‐DQ602*) is the major protective haplotype in European‐derived populations acting dominantly also when paired with a high‐risk haplotype [5, 6]. Indeed, up to 90% of patients with T1D carry one of the risk haplotypes and less than 20% develop overt disease with neutral or protective HLA class II genotypes. Conversely, DQB1*06:02 has been reported to be carried by less than 1% of patients with T1D, while its prevalence in the general population is approximately 15% [5, 7, 8]. Although DQB1*06:02 does not provide complete protection from T1D, it is associated with decreased frequencies of diabetes‐associated autoantibodies. Moreover, protective effects have also been detected after seroconversion to positivity for one or more of these autoantibodies, leading to less likely progression from the prediabetic phase to overt disease [9, 10, 11].

Several studies have shown that patients with higher‐risk HLA genotypes present with T1D earlier in life, whereas the frequency of protective haplotypes is higher among patients diagnosed at an older age [12, 13, 14]. We also recently reported heterogeneity in relation to clinical and humoral markers at diagnosis of T1D depending on the HLA class II genotype [15, 16].

Here, we set out to analyse whether carrying the protective DQB1*06:02 allele would be associated with distinct characteristics of T1D at the time of clinical diagnosis. We compared children with T1D carrying this allele to those with any other genotype and those with a high‐risk genotype. Furthermore, we assessed whether there were any differences in the characteristics of T1D and whether patients carrying the protective haplotype also carried a haplotype conferring high disease susceptibility or not. We analysed clinical, demographic, metabolic and humoral characteristics. We hypothesized that children with T1D carrying the protective haplotype would be diagnosed at an older age and would have a less aggressive disease manifestation.

## 2 Study Design and Methods

### 2.1 Study Population

Our data are derived from the Finnish Pediatric Diabetes Register (FDPR), which is a national register established in 2002. The register has a wide coverage, accounting for more than 90% of all new paediatric patients diagnosed with T1D [17]. Inclusion criteria for participants in the current study were (i) age under 15 years, (ii) diagnosed with T1D between 2003 and 2018, (iii) samples available for autoantibody analysis and taken within 31 days of diagnosis, (iv) no suspicion of monogenic T1D (i.e., age under 1 year, a protective HLA genotype and no positive autoantibodies), (v) informed parental consent to their children's participation and (vi) HLA data available. Children aged 10 years or older were also asked for informed consent. T1D was diagnosed in 7894 children under 15 years of age within the observed time frame. Information on HLA class II genotypes was available for 77.1% of the children with T1D and based on the inclusion criteria the final number of children with T1D was 5530. The Ethics Committee of the Hospital District of Helsinki and Uusimaa has approved the study protocol (identification number 457/E7/2001).

The children with T1D were divided into groups depending on their HLA class II genotype. Children with T1D carrying the protective DQB1*06:02 allele (*N* = 75) were compared to children with T1D with any other genotype (*N* = 5455) and those with a high‐risk genotype (*N* = 1182) according to the risk classification presented by Ilonen et al. in 2016 [6]. In addition, we divided children with T1D carrying the DQB1*06:02 allele into those with a risk‐associated haplotype pair (*N* = 51) and those without such a haplotype (*N* = 24). The risk‐conferring susceptibility haplotypes were DRB1*04:01/02/04/05‐DQA1*03‐DQB1*03:02 (*DR4‐DQ8*) and DRB1*03:01‐DQA1*05‐DQB1*02 (*DR3‐DQ2*).

### 2.2 Clinical and Demographic Markers

Demographic factors analysed were age at diagnosis, sex and family history. We had information on T1D diagnoses among first‐degree relatives. If there were multiple affected siblings in the same family, only the sibling who was diagnosed first was included as an index child. We assessed several clinical and metabolic markers of T1D at the time of diagnosis: blood pH and haemoglobin A1c (HbA1c) as well as glucose and β‐hydroxybutyrate from plasma. Blood pH levels were measured in 97% of children with T1D, and according to the pH levels, we compared the frequencies of ketoacidosis (pH < 7.30) and severe ketoacidosis (pH < 7.10). The registration of the HbA1c levels started in 2013, which explains why only 28% of the children with T1D had available data. Body mass index standard deviation scores (BMI SDS) were calculated based on the children's heights and weights at the time of diagnosis using the World Health Organization AnhtroPlus software [18]. Duration of symptoms was assessed with a questionnaire completed by the children's parents.

### 2.3 Disease‐Associated Autoantibodies and HLA Genotyping

We analysed the frequencies of positivity and levels of five diabetes‐associated autoantibodies including islet cell antibodies (ICAs), insulin autoantibodies (IAAs), glutamic acid decarboxylase autoantibodies (GADAs), islet antigen 2 autoantibodies (IA‐2As) and zinc transporter 8 autoantibodies (ZnT8As). The biochemical autoantibodies (ICA excluded) were measured with specific radiobinding assays as previously described [19], while ICA was analysed with indirect immunofluorescence on pancreata from human group 0 donors [20]. The cut‐off limits for positivity for each autoantibody were as follows: 1.57 relative units (RU) for IAA, 0.77 RU for IA‐2A, 5.36 RU for GADA and 0.50 RU for ZnT8A. For ICA, the cut‐off limit was 2.5 Juvenile Diabetes Research Foundation (JDRF) units. The sensitivities and specificities of the radiobinding assays during 2003–2018 were 42%–66% and 92%–99% for IAA, 62%–72% and 93%–100% for IA‐2A, 64%–90% and 90%–99% for GADA, 62%–70% and 99%–100% for ZnT8A. According to a previous report [21], it is possible to estimate the initially seroconverted autoantibody based on the autoantibody profile at the time of diagnosis. GADA appearing alone or combined with other biochemical autoantibodies other than IAA can be categorized in the “GADA‐first” group and vice versa for IAA. When comparing autoantibody titres, only those with positive levels were included.

The HLA genotyping was performed with PCR‐based lanthanide‐labelled hybridization followed by time‐resolved fluorometry detection. The methods have been described in detail previously [22].

### 2.4 Statistical Analysis

We used R software (version 4.2.2) for all statistical analyses. Categorical variables were reported as frequencies and proportions, skewed continuous variables as median and range and normally distributed variables as mean and standard deviation (SD). Cross tabulation and Pearson's *χ* ^2^ test, with continuity correction when appropriate, were used to compare frequencies between different groups. Normally distributed variables were compared with Student's *t*‐test while nonparametric variables were analysed with the Mann–Whitney *U* test. We adjusted the results for age and sex as we saw significant differences in the age at diagnosis between the examined groups and previous studies show that the characteristics of T1D vary between sexes [23]. The adjustments were performed with generalized/ordinal logistic regression for categorical variables and quantile regression (quantreg package in R) for continuous variables. Because of the lower sample sizes in the analyses of Tables 2 and 3, we implemented the bootstrapping method in some of the analyses with quantile regression to obtain *p* values. The adjusted analyses were corrected for multiple testing using the Benjamini–Hochberg procedure. A *p* value < 0.05 was considered to indicate statistical significance.

## 3 Results

### 3.1 Demographics

Of all the children with T1D 75 (1.36%) carried the protective DQB1*06:02 allele, the majority were male in all groups studied, but we observed no significant differences in the sex distribution (Tables 1, 2, 3). Children with T1D carrying DQB1*06:02 were diagnosed at an older age than the children with T1D carrying other HLA class II genotypes (9.48 years vs. 8.07 years; *p* = 0.003). Children carrying a high‐risk genotype were diagnosed even younger, that is, on an average at the age of 7.70 years (*p* < 0.001). No significant differences in the age at diagnosis could be detected when comparing children with T1D carrying the DQB1*06:02 allele with or without a risk‐associated haplotype (Table 3). No significant differences in the frequencies of affected family members were observed, although carriers of the DQB1*06:02 positive haplotype had no affected fathers when compared to 5.5% among children with T1D carrying other genotypes (*p* = 0.068, adjusted *p* = 0.96).

**TABLE 1:** Comparison of demographic, metabolic and immunological markers at diagnosis between children with T1D carrying a DQB1*06:02‐positive protective HLA class II haplotype and all other participants.

Variable	*N*	DQB1*06:02‐positive (*N* = 75)	Other (*N* = 5455)	*p*	Adjusted *p*
Sex, male, *N* (%)	5042	42 (56.0)	3085 (56.6)	1	
Age at diagnosis, years, mean ± SD	5530	9.48 ± 3.98	8.07 ± 3.87	**0.003**	
Family history of type 1 diabetes	5530				
Father with type 1 diabetes, %		0.0	5.5	0.068	0.96
Mother with type 1 diabetes, %		5.3	3.0	0.41	0.19
Sibling with type 1 diabetes, %		6.7	6.3	1.00	0.77
Family member with type 1 diabetes, %		10.7	13.9	0.53	0.56
BMI SDS, *z*‐score mean, ± SD	5291	−0.34 ± 1.49	−0.22 ± 1.37	0.48	0.60
pH, median (range)	5351	7.38 (6.96–7.49)	7.38 (6.69–7.57)	0.38	0.91
Ketoacidosis, %	5351	21.6	18.7	0.63	0.79
Severe ketoacidosis, %	5351	2.7	5.0	0.53	0.28
β‐hydroxybutyrate, mmol/L, median (range)	4884	1.43 (0–15.10)	1.77 (0–52.00)	0.79	0.44
Plasma glucose, mmol/L, median (range)	5397	24.95 (6.00–72.10)	23.90 (3.20–95.60)	0.14	0.33
HbA1c, mmol/mol, median (range)	1523	106.00 (75.00–153.00)	91.26 (30.00–189.00)	**0.014**	0.78
HbA1c, %, median (range)	1523	11.85 (9.01–16.15)	10.50 (4.89–19.44)	**0.014**	0.79
Duration of symptoms	5068			0.38	0.98
No symptoms, %		0	1.5		
< 1 week, %		17.6	22.2		
1–2 weeks, %		35.1	32.0		
2–3 weeks, %		6.8	11.0		
3–4 weeks, %		20.3	14.0		
> 4 weeks, %		20.3	19.2		
Frequency of autoantibodies					
IAA positivity, %	5530	56.0	55.1	0.97	0.15
IA‐2A positivity, %	5530	76.0	74.6	0.89	0.82
GADA positivity, %	5530	76.0	66.1	0.093	0.11
ZnT8A positivity, %	5528	72.0	69.4	0.71	0.79
ICA positivity, %	5528	92.2	91.3	0.97	0.62
Autoantibody levels					
IAA, RU, median (range)	3048	4.67 (1.71–116.58)	7.07 (1.57–7809.00)	0.038	0.91
IA‐2A, RU, median (range)	4126	94.28 (1.06–266.52)	105.24 (0.78–553.32)	0.12	0.27
GADA, RU, median (range)	3662	53.79 (6.01–206.03)	35.58 (5.36–24849.00)	0.24	**0.033**
ZnT8A, RU, median (range)	3829	13.70 (0.54–141.44)	11.83 (0.51–1201.90)	0.97	0.87
ICA, JDRF, median (range)	5026	65.00 (6.00–1025.00)	32.00 (2.00–5120.00)	1.00	0.92
Initial autoantibody	5530				
GADA		32.0	26.9	0.39	0.95
IAA		12.0	15.9	0.45	0.76
Sum of autoantibodies, ICA excluded	5530			0.79	0.55
0, %		2.7	3.5		
1, %		8.0	12.2		
2, %		25.3	25.4		
3, %		34.7	33.7		
4, %		29.3	25.2		

*Note:* Correction for multiple testing was carried out using the Benjamini–Hochberg procedure. All false discovery rate (FDR)‐corrected *p* values in Table 1 were > 0.05. The bolded values are significant (*p* < 0.05).

Abbreviations: BMI SDS, body mass index standard deviation scores; HbA1c, haemoglobin A1c; IA‐2A, islet antigen 2 autoantibodies; IAA, insulin autoantibodies; ICA, islet cell antibodies; GADA, glutamic acid decarboxylase autoantibodies; JDRF, Juvenile Diabetes Research Foundation units; RU, relative units; SD, standard deviation; ZnT8A, zinc transporter 8 autoantibodies.

**TABLE 2:** Comparison of demographic, metabolic and immunological markers at diagnosis between children with T1D carrying a DQB1*06:02‐positive protective HLA class II haplotype and patients carrying high‐risk HLA class II genotype.

Variable	*N*	DQB1*06:02‐positive (*N* = 75)	High‐risk genotype (*N* = 1182)	*p*	Adjusted *p*
Sex, male, *N* (%)	1257	42 (56.0)	663 (56.1)	1	
Age at diagnosis, years, mean ± SD	1257	9.48 ± 3.98	7.70 ± 3.96	**< 0.001**	
Family history of type 1 diabetes	1257				
Father with type 1 diabetes, %		0.0	5.1	0.085	0.98
Mother with type 1 diabetes, %		5.3	3.0	0.45	0.19
Sibling with type 1 diabetes, %		6.7	8.3	0.78	0.70
Family member with type 1 diabetes, %		10.7	15.4	0.35	0.35
BMI SDS, *z*‐score mean, ± SD	1203	−0.34 ± 1.49	−0.19 ± 1.31	0.41	0.91
pH, median (range)	1208	7.38 (6.96–7.49)	7.38 (6.79–7.54)	0.18	0.71
Ketoacidosis, %	1208	21.6	16.2	0.29	0.29
Severe ketoacidosis, %	1208	2.7	3.9	0.84	0.53
β‐Hydroxybutyrate, mmol/L, median (range)	1088	1.43 (0–15.10)	1.60 (0–18.00)	0.72	0.71
Plasma glucose, mmol/L, median (range)	1223	24.95 (6.00–72.10)	23.60 (3.60–81.00)	**0.050**	0.10
HbA1c, mmol/mol, median (range)	360	106.00 (75.00–153.00)	87.98 (36.00–165.00)	**0.004**	0.60
HbA1c, %, median (range)	360	11.85 (9.01–16.15)	10.20 (5.44–17.25)	**0.004**	0.61
Duration of symptoms	1151			0.37	0.99
No symptoms, %		0	1.9		
< 1 week, %		17.6	24.4		
1–2 weeks, %		35.1	31.6		
2–3 weeks, %		6.8	9.8		
3–4 weeks, %		20.3	14.9		
> 4 weeks, %		20.3	17.4		
Frequency of autoantibodies					
IAA positivity, %	1257	56.0	61.3	0.43	0.45
IA‐2A positivity, %	1257	76.0	74.0	0.81	0.74
GADA positivity, %	1257	76.0	69.9	0.32	0.46
ZnT8A positivity, %	1254	72.0	64.2	0.21	0.79
ICA positivity, %	1257	92.0	90.1	0.74	0.43
Autoantibody levels					
IAA, RU, median (range)	767	4.67 (1.71–116.58)	7.92 (1.57–309.25)	**0.012**	0.68
IA‐2A, RU, median (range)	932	94.28 (1.06–266.52)	99.09 (0.80–254.05)	0.43	0.36
GADA, RU, median (range)	883	53.79 (6.01–206.03)	42.14 (5.52–10241.86)	0.91	0.99
ZnT8A, RU, median (range)	811	13.70 (0.54–141.44)	9.47 (0.51–1201.90)	0.46	0.42
ICA, JDRF, median (range)	1134	65.00 (6.00–1025.00)	49.00 (3.00–5120.00)	0.23	0.066
Initial autoantibody	1257				
GADA		32.0	25.2	0.24	0.88
IAA		12.0	16.7	0.37	0.83
Sum of autoantibodies, ICA excluded	1257			0.88	0.62
0, %		2.7	3.2		
1, %		8.0	11.8		
2, %		25.3	25.0		
3, %		34.7	32.2		
4, %		29.34	27.7		

*Note:* Correction for multiple testing was carried out using the Benjamini–Hochberg procedure. All false discovery rate (FDR)‐corrected *p* values in Table 2 were > 0.05. The bolded values are significant (*p* < 0.05).

Abbreviations: BMI SDS, body mass index standard deviation scores; HbA1c, haemoglobin A1c; IA‐2A, islet antigen 2 autoantibodies; IAA, insulin autoantibodies; ICA, islet cell antibodies; GADA, glutamic acid decarboxylase autoantibodies; JDRF, Juvenile Diabetes Research Foundation units; RU, relative units; SD, standard deviation; ZnT8A, zinc transporter 8 autoantibodies.

**TABLE 3:** Comparison of demographic, metabolic and immunological markers at diagnosis between children with T1D carrying the DQB1*06:02‐positive protective haplotype ± a high‐risk haplotype.

Variable	*N*	DQB1*06:02‐positive + high‐risk haplotype (*N* = 51)		DQB1*06:02‐positive without high‐risk haplotype (*N* = 24)	*p*	Adjusted *p*
Sex, male, *N* (%)	75	29 (56.9)		13 (54.2)	1.00	
Age at diagnosis, years, mean ± SD	75	9.60 ± 3.91		9.22 ± 4.21	0.71	
Family history of type 1 diabetes	75					
Father with type 1 diabetes, %		0		0	—	—
Mother with type 1 diabetes, %		5.9		4.2	1.0	0.73
Sibling with type 1 diabetes, %		7.8		4.2	0.92	0.55
Family member with type 1 diabetes, %		11.8		8.3	0.96	0.62
BMI SDS, *z*‐score mean, ± SD	73	−0.45 ± 1.45		−0.11 ± 1.57	0.37	0.55
pH, median (range)	74	7.36 (7.12–7.49)		7.39 (6.96–7.43)	0.36	0.079
Ketoacidosis, %	74	25.5		13.0	0.37	0.26
Severe ketoacidosis, %	74	0.0		8.7	0.17	1.00
β‐Hydroxybutyrate, mmol/L, median (range)	66	1.58 (0–15.10)		1.10 (0.09–7.62)	0.32	0.64
Plasma glucose, mmol/L, median (range)	74	25.20 (15.80–72.10)		24.70 (6.00–54.30)	0.83	0.49
HbA1c, mmol/mol, median (range)	25	97.50 (82.00–153.00)		107.00 (75.00–152.00)	0.81	0.87
HbA1c, %, median (range)	25	11.07 (9.65–16.15)		11.94 (9.01–16.06)	0.81	0.87
Duration of symptoms	74				0.19	**0.043**
No symptoms, %		0.0		0.0		
< 1 week, %		24.0		4.2		
1–2 weeks, %		36.0		33.3		
2–3 weeks, %		4.0		12.5		
3–4 weeks, %		18.0		25.0		
> 4 weeks, %		18.0		25.0		
Frequency of autoantibodies						
IAA positivity, %	75	54.9		58.3	0.98	0.83
IA‐2A positivity, %	75	78.4		70.8	0.67	0.48
GADA positivity, %	75	78.4		70.8	0.67	0.53
ZnT8A positivity, %	75	76.5		62.5	0.33	0.24
ICA positivity, %	75	92.2		91.7	1.00	0.94
Autoantibody levels						
IAA, RU, median (range)	31	4.34 (1.72–67.58)		6.10 (1.71–116.58)	0.50	0.90
IA‐2A, RU, median (range)	57	114.22 (2.25–266.52)		25.29 (1.06–147.58)	**0.005**	**< 0.001**
GADA, RU, median (range)	57	51.03 (6.01–206‐03)		54.87 (17.31–194.10)	0.41	0.78
ZnT8A, RU, median (range)	54	9.84 (0.55–141.44)		25.06 (0.54–88.43)	0.81	0.36
ICA, JDRF, median (range)	69	96.00 (6.00–1025.00)		47.00 (7.00–512.00)	**0.028**	0.15
Initial autoantibody						
GADA	75	35.3		25.0	0.53	0.40
IAA	65	11.8		12.5	1.00	0.86
Sum of autoantibodies, ICA excluded	75				0.086	1.00
0, %		0.0		8.3		
1, %		5.9		12.5		
2, %		31.4		12.5		
3, %		31.4		41.7		
4, %		31.4		25.0		

*Note:* Correction for multiple testing was carried out using the Benjamini–Hochberg procedure. All false discovery rate (FDR)‐corrected *p* values in Table 3 were > 0.05 except for the comparison of the IA‐2A titres (*p* = 0.001). The bolded values are significant (*p* < 0.05).

Abbreviations: BMI SDS, body mass index standard deviation scores; HbA1c, haemoglobin A1c; IA‐2A, islet antigen 2 autoantibodies; IAA, insulin autoantibodies; ICA, islet cell antibodies; GADA, glutamic acid decarboxylase autoantibodies; JDRF, Juvenile Diabetes Research Foundation units; RU, relative units; SD, standard deviation; ZnT8A, zinc transporter 8 autoantibodies.

### 3.2 Clinical and Metabolic Characteristics

In the initial analyses, the carriers of DQB1*06:02 allele had higher glucose levels compared to carriers of the high‐risk genotype (median 24.95 mmol/L vs. 23.60 mmol/L, *p* = 0.050). Similarly, HbA1c levels were higher among carriers of the protective haplotype when compared to carriers of other genotypes (106.00 mmol/mol; 11.85% vs. 91.26 mmol/mol, 10.50%, *p* = 0.014) and those with a high‐risk genotype (87.98 mmol/mol, 10.20%, *p* = 0.004). However, adjusting the results for age and sex removed any significance indicating that the differences observed in clinical and metabolic characteristics were due to age and sex variations (Tables 1 and 2). There were no significant differences in levels of pH or β‐hydroxybutyrate at the time of diagnosis. Accordingly, no differences in the frequencies of ketoacidosis or severe ketoacidosis were observed.

Pairing a high‐risk haplotype to a DQB1*06:02‐positive haplotype did not affect metabolic markers at the time of diagnosis, although children with T1D carrying that haplotype combination had a somewhat shorter duration of symptoms (adjusted *p* = 0.043, FDR corrected *p* value 0.54) (Table 3).

### 3.3 Beta‐Cell Autoimmunity

No significant differences in the frequencies of autoantibody positivity were observed in our analyses. However, GADA levels were significantly higher among carriers of DQB1*06:02 compared to carriers of other HLA class II genotypes (53.79 RU vs. 35.58 RU, *p* = 0.24, adjusted *p*  = 0.033). Vice versa, in the initial analyses, patients with other genotypes (7.07 RU vs. 4.67 RU, *p* = 0.038, adjusted *p* = 0.91) or the high‐risk genotype (7.92 RU vs. 4.67 RU, *p* = 0.012, adjusted *p* = 0.68) had higher levels of IAA than carriers of DQB1*06:02 (Tables 1 and 2).

The carriers of DQB1*06:02 had more often GADA as the first appearing autoantibody compared to carriers of other HLA class II genotypes (32.0% vs. 26.9%, *p* = 0.39, adjusted *p* = 0.95) and those with a high‐risk genotype (32.0% vs. 25.2%, *p* = 0.24, adjusted  *p* = 0.88). These differences remained, however, nonsignificant (Tables 1 and 2).

When looking at children with T1D carrying DQB1*06:02 with or without a high‐risk haplotype, no significant differences in autoantibody frequencies were detected. However, the comparison of these groups revealed higher levels of IA‐2A (114.22 RU vs. 25.29 RU, *p* = 0.005, adjusted *p* < 0.001, FDR corrected *p* value = 0.001) among children with T1D with a high‐risk haplotype combination (Table 3).

## 4 Discussion

In this cross‐sectional survey based on a nationwide register, we found some differences in the characteristics of T1D depending on whether the affected patient carried the protective HLA‐DQB1*06:02 allele or not. Based on previous reports we expected that children with T1D with DQB1*06:02 would be diagnosed at an older age when compared to those with other HLA class II genotypes or the high‐risk genotype. Indeed, we observed that the higher the risk conferred by the HLA genotype, the younger the age at diagnosis was. Male sex was more frequent in all groups independently of the HLA genotype. This is in line with previous studies which have shown a positive association between male sex and the T1D incidence in countries with high incidence of T1D [24].

In relation to clinical and metabolic markers, we initially observed significant differences in plasma glucose concentrations and HbA1c levels at the time of diagnosis. Contrary to our expectations, the median plasma glucose was higher among carriers of the DQB1*06:02 allele when compared to those with a high‐risk genotype. Similarly, children with T1D with DQB1*06:02‐positive haplotype had higher HbA1c values when compared to patients with other or high‐risk HLA genotypes. However, adjusting for age and sex rendered these findings statistically insignificant. These effects of age and sex especially on HbA1c are in line with previous studies. Extensive registry‐based studies both in Sweden and Finland have shown that girls have higher HbA1c levels at the time of diagnosis [25, 26, 27].

The disease process leading to clinical T1D may take years. It has been estimated that the clinical manifestation of the disease occurs when only 10%–30% of the insulin‐producing beta cells are left functioning [28]. Age at diagnosis could be a more comprehensive marker of the aggressivity of the autoimmune process than metabolic markers that are affected more by what happens in the end‐stage of the disease process. The metabolic markers might more likely be affected by social factors and the availability of healthcare than the velocity of the disease process at the verge of diagnosis. Thus, carrying protective haplotypes could indirectly affect manifestation of T1D at the time of diagnosis by delaying the disease presentation. Indeed, Pugliese et al. [11] have previously argued how adjusting for age would misleadingly diminish the impact of carrying the DQB1*06:02 allele as older age at the time of diagnosis is a likely outcome of its protective properties.

There were no significant differences in the frequency of autoantibody positivity, but we observed differences in GADA titres depending on the HLA class II genotype. Children with T1D with the protective genotype had significantly higher GADA titres than those with other genotypes. This may be related to the increased frequency of the DRB1*04:04‐DQA1*03‐DQB1*0302 haplotype as the second haplotype in DQB1*06:02 positive children with T1D [29]. In a previous study, we have shown that autoantibodies against the GAD M‐epitope and C‐epitope were more common among those T1D children carrying the DRB1*04:04 allele than among those negative for this allele [30]. Preferential binding to certain islet peptide epitopes by the DR404 but not the DR401 molecule to be presented to effector T cells could be hypothesized as a mechanism behind our observation of the increased DRB1*04:04 allele frequency and higher GADA titres found in DQB1*0602‐positive children with T1D.

Before adjusting the results, we could also see higher levels of IAA among carriers of other HLA genotypes or of the high‐risk genotype. Recently, our understanding of T1D has shifted towards the notion of disease heterogeneity and different endotypes of type 1 diabetes, which could be classified in two main endotypes. The first one is associated with faster disease progression rate and is characterized by IAA as the first autoantibody to emerge in the preclinical phase peaking as early as around the age of 1–2 years. The second endotype is associated with initial seroconversion of GADA with a lower peak 2–3 years later in life [31]. We observed that GADA was the more frequent and IAA the more infrequent initial autoantibody among carriers of the DQB1*06:02 allele but no significant differences could be detected. It must be noted that significant reverse seroconversions before the diagnosis of T1D have been observed for IAA, with the probability of this phenomenon increasing with the duration of the prediabetic period [31]. As children with T1D in our study are up to 14‐year‐old, inverse IAA seroconversions may affect the analysis of IAA frequencies and levels.

To compare patients carrying DQB1*06:02 to those carrying other HLA class II genotypes, we also performed within‐group analyses in the protective group. As mentioned in the introduction, DQB1*06:02 has been shown to offer dominant protection from T1D even when paired with a haplotype conferring strong disease susceptibility. Here, we set out to assess whether there might be any differences in the presentation of T1D depending on whether the haplotype paired with the DQB1*06:02‐positive one conferred high risk or not. We observed a longer duration of symptoms among patients without a risk‐associated haplotype, but no other significant effects on clinical, demographic or metabolic factors at diagnosis could be seen. Neither did we observe any differences in the frequencies of autoantibodies. However, we detected markedly higher concentrations of IA‐2A among children with T1D carrying a risk‐associated haplotype. The high titre of IA‐2A is fitting since carrying a genotype including the DRB1*04‐DQB1*03:02 haplotype has previously been associated with the highest levels of IA‐2A [32]. IA‐2A has been suggested to be a more specific marker of beta‐cell autoimmunity because of its association with the high‐risk haplotype, low serum C‐peptide levels at diagnosis and higher need for exogenous insulin during the first years of overt disease [32]. However, no clinical indication of a more advanced or aggressive disease was detected here.

### 4.1 Strengths and Limitations

A major strength of our study is the large sample size since our study is based on a national register with a coverage of more than 90% in the country with the highest incidence of T1D globally. As far as we are aware, the current DQB1*06:02 cohort is the largest one analysed so far. When considering limitations, the cross‐sectional design prevents us from observing the progression of the disease process before the time of diagnosis. Information on the characteristics preceding the diagnosis of T1D would reflect the aggressiveness of the disease more in detail. As expected the number of patients carrying DQB1*06:02 allele is rather low, increasing the likelihood of type two errors when comparing characteristics of T1D within the group. We do not have information on non‐HLA susceptibility genes for T1D which might explain some findings or the lack thereof. Finally, the Finnish population is very homogenous which should be taken into account when generalizing these results.

## 5 Conclusions

Our findings are in line with previous reports regarding the association between HLA class II genotypes and age at diagnosis: the association is evident as carriers of DQB1*06:02 are diagnosed at the oldest age and carriers of high‐risk genotypes at the youngest one. Differences in the clinical manifestation seem to be explained primarily by other factors than the HLA class 2 genotype. Our results support previous reports regarding the dominant effects of the DQB1*06:02‐positive haplotype as pairing the protective haplotype with a high‐risk haplotype had only minor effects on the characteristics of T1D.

## Author Contributions

Conceptualization and methodology, funding acquisition and resources: M.K., T.H., R.V. and J.I. Data analysis and software: A.T.M., T.V. and M.T.V. Supervision, sampling and laboratory tests: T.H., P.V., J.L. and J.I. Data validation, reviewing and editing the manuscript: all authors.

## Conflicts of Interest

The authors declare no conflicts of interest.

## Supporting information


**Appendix S1.** Supporting Information.

## Data Availability

The data that support the findings of this study are available on request from the corresponding author. The data are not publicly available due to privacy or ethical restrictions.

## References

[1] M. Karvonen , M. Viik‐Kajander , E. Moltchanova , I. Libman , R. LaPorte , and J. Tuomilehto , “Incidence of Childhood Type 1 Diabetes Worldwide,” Diabetes Care 23, no. 10 (2000): 1516–1526, 10.2337/diacare.23.10.1516. 11023146

[2] J. A. Todd , J. I. Bell , and H. O. McDevitt , “Hla‐Dq Beta Gene Contributes to Susceptibility and Resistance to Insulin‐Dependent Diabetes Mellitus,” Nature 329, no. 6140 (1987): 599–604, 10.1038/329599a0. 3309680

[3] J. A. Noble and A. M. Valdes , “Genetics of the Hla Region in the Prediction of Type 1 Diabetes,” Current Diabetic Reports 11, no. 6 (2011): 533–542, 10.1007/s11892-011-0223-x. PMC3233362 21912932

[4] C. C. Robertson , J. R. J. Inshaw , S. Onengut‐Gumuscu , et al., “Fine‐Mapping, Trans‐Ancestral And Genomic Analyses Identify Causal Variants, Cells, Genes And Drug Targets for Type 1 Diabetes,” Nature Genetics 53, no. 7 (2021): 962–971, 10.1038/s41588-021-00880-5. 34127860 PMC8273124

[5] H. Erlich , A. M. Valdes , J. Noble , et al., “Hla Dr‐Dq Haplotypes and Genotypes and Type 1 Diabetes Risk: Analysis of the Type 1 Diabetes Genetics Consortium Families,” Diabetes 57, no. 4 (2008): 1084–1092, 10.2337/db07-1331. 18252895 PMC4103420

[6] A. Michels , L. Zhang , A. Khadra , J. A. Kushner , M. J. Redondo , and M. Pietropaolo , “Prediction and Prevention of Type 1 Diabetes: Update on Success of Prediction and Struggles at Prevention,” Pediatric Diabetes 16, no. 7 (2015): 465–484, 10.1111/pedi.12299. 26202050 PMC4592445

[7] M. J. Redondo , A. K. Steck , and A. Pugliese , “Genetics of Type 1 Diabetes,” Pediatric Diabetes 19, no. 3 (2018): 346–353, 10.1111/pedi.12597. 29094512 PMC5918237

[8] W. Klitz , M. Maiers , S. Spellman , et al., “New Hla Haplotype Frequency Reference Standards: High‐Resolution and Large Sample Typing of Hla Dr‐Dq Haplotypes in a Sample of European Americans,” Tissue Antigens 62, no. 4 (2003): 296–307, 10.1034/j.1399-0039.2003.00103.x. 12974796

[9] A. Pugliese , R. Gianani , R. Moromisato , et al., “Hla‐DQB1*0602 Is Associated With Dominant Protection From Diabetes Even Among Islet Cell Antibody‐Positive First‐Degree Relatives of Patients With IDDM,” Diabetes 44, no. 6 (1995): 608–613, 10.2337/diab.44.6.608. 7789622

[10] C. J. Greenbaum , D. A. Schatz , D. Cuthbertson , A. Zeidler , G. S. Eisenbarth , and J. P. Krischer , “Islet Cell Antibody‐Positive Relatives With Human Leukocyte Antigen DQA1*0102, DQB1*0602: Identification by the Diabetes Prevention Trial‐Type 1,” Journal of Clinical Endocrinology and Metabolism 85, no. 3 (2000): 1255–1260, 10.1210/jcem.85.3.6459. 10720072

[11] A. Pugliese , D. Boulware , L. Yu , et al., “Hla‐DRB1*15:01‐DQA1*01:02‐DQB1*06:02 Haplotype Protects Autoantibody‐Positive Relatives From Type 1 Diabetes Throughout the Stages of Disease Progression,” Diabetes 65, no. 4 (2016): 1109–1119, 10.2337/db15-1105. 26822082 PMC4806662

[12] A. M. Valdes , H. A. Erlich , J. Carlson , M. Varney , P. V. Moonsamy , and J. A. Noble , “Use of Class I and Class Ii Hla Loci for Predicting Age At Onset of Type 1 Diabetes In Multiple Populations,” Diabetologia 55, no. 9 (2012): 2394–2401, 10.1007/s00125-012-2608-z. 22706720 PMC3639291

[13] K. M. Gillespie , E. A. Gale , and P. J. Bingley , “High Familial Risk and Genetic Susceptibility in Early Onset Childhood Diabetes,” Diabetes 51, no. 1 (2002): 210–214, 10.2337/diabetes.51.1.210. 11756343

[14] J. R. J. Inshaw , A. J. Cutler , D. J. M. Crouch , L. S. Wicker , and J. A. Todd , “Genetic Variants Predisposing Most Strongly to Type 1 Diabetes Diagnosed Under Age 7 Years Lie Near Candidate Genes That Function in the Immune System and in Pancreatic Β‐Cells,” Diabetes Care 43, no. 1 (2020): 169–177, 10.2337/dc19-0803. 31558544 PMC6925581

[15] A. M. Taka , T. Härkönen , P. Vähäsalo , et al., “Heterogeneity in the Presentation of Clinical Type 1 Diabetes Defined by the Level Of Risk Conferred by Human Leukocyte Antigen Class II Genotypes,” Pediatric Diabetes 23, no. 2 (2022): 219–227, 10.1111/pedi.13300. 34894365

[16] M. L. Mikk , S. Pfeiffer , M. Kiviniemi , et al., “Hla‐DR‐DQ Haplotypes and Specificity of the Initial Autoantibody in Islet Specific Autoimmunity,” Pediatric Diabetes 21, no. 7 (2020): 1218–1226, 10.1111/pedi.13073. 32613719

[17] A. Hekkala , A. Reunanen , M. Koski , M. Knip , and R. Veijola , “Age‐Related Differences in the Frequency of Ketoacidosis At Diagnosis of Type 1 Diabetes in Children and Adolescents,” Diabetes Care 33, no. 7 (2010): 1500–1502, 10.2337/dc09-2344. 20413519 PMC2890349

[18] World Health Organization , “AnthroPlus for Personal Computers Manual: Software for Assessing Growth of the World's Children and Adolescents,” accessed March 29, 2021, https://www.who.int/growthref/tools/en/.

[19] M. Knip , S. M. Virtanen , K. Seppä , et al., “Dietary Intervention in Infancy and Later Signs of Beta‐Cell Autoimmunity,” New England Journal of Medicine 363, no. 20 (2010): 1900–1908, 10.1056/NEJMoa1004809. 21067382 PMC4242902

[20] G. F. Bottazzo , A. Florin‐Christensen , and D. Doniach , “Islet‐Cell Antibodies in Diabetes Mellitus With Autoimmune Polyendocrine Deficiencies,” Lancet 2, no. 7892 (1974): 1279–1283, 10.1016/s0140-6736(74)90140-8. 4139522

[21] J. Ilonen , A. P. Laine , M. Kiviniemi , T. Härkönen , J. Lempainen , and M. Knip , “Associations Between Deduced First Islet Specific Autoantibody With Sex, Age at Diagnosis And Genetic Risk Factors in Young Children With Type 1 Diabetes,” Pediatric Diabetes 23, no. 6 (2022): 693–702, 10.1111/pedi.13340. 35403376 PMC9541564

[22] M. L. Mikk , M. Kiviniemi , A. P. Laine , et al., “The Hla‐B*39 Allele Increases Type 1 Diabetes Risk Conferred By Hla‐Drb1*04:04‐Dqb1*03:02 And Hla‐Drb1*08‐Dqb1*04 Class Ii Haplotypes,” Human Immunology 75, no. 1 (2014): 65–70, 10.1016/j.humimm.2013.09.008. 24055898

[23] S. A. G. de Vries , C. L. Verheugt , D. Mul , M. Nieuwdorp , and T. C. J. Sas , “Do Sex Differences in Paediatric Type 1 Diabetes Care Exist? A systematic review,” Diabetologia 66, no. 4 (2023): 618–630, 10.1007/s00125-022-05866-4. 36700969 PMC9947056

[24] M. Karvonen , M. Pitkäniemi , J. Pitkäniemi , K. Kohtamäki , N. Tajima , and J. Tuomilehto , “Sex Difference in the Incidence of Insulin‐Dependent Diabetes Mellitus: An Analysis of the Recent Epidemiological Data,” Diabetes/Metabolism Reviews 13, no. 4 (1997): 275–291, 10.1002/(sici)1099-0895(199712)13:4<275::aid-dmr197>3.0.co;2-v. 9509279

[25] L. Hanberger , K. Åkesson , and U. Samuelsson , “Glycated Haemoglobin Variations in Paediatric Type 1 Diabetes: The Impact of Season, Gender and Age,” Acta Paediatrica 103, no. 4 (2014): 398–403, 10.1111/apa.12530. 24299617

[26] K. Åkesson , L. Hanberger , and U. Samuelsson , “The Influence of Age, Gender, Insulin Dose, Bmi, and Blood Pressure on Metabolic Control in Young Patients With Type 1 Diabetes,” Pediatric Diabetes 16, no. 8 (2015): 581–586, 10.1111/pedi.12219. 25270077

[27] M. Turtinen , T. Härkönen , A. Parkkola , J. Ilonen , and M. Knip , “Sex as a Determinant of Type 1 Diabetes At Diagnosis,” Pediatric Diabetes 19, no. 7 (2018): 1221–1228, 10.1111/pedi.12697. 29862628

[28] M. Knip , “Disease‐Associated Autoimmunity and Prevention of Insulin‐Dependent Diabetes Mellitus,” Annals of Medicine 29, no. 5 (1997): 447–451, 10.3109/07853899708999375. 9453293

[29] J. Ilonen , M. Kiviniemi , M. I. El‐Amir , et al., “Increased Frequency of the Hla‐DRB1*04:04‐DQA1*03‐DQB1*03:02 Haplotype Among Hla‐Dqb1*06:02 Positive Children With Type 1 Diabetes,” Diabetes 73, no. 2 (2024): 306–311, 10.2337/db23-0387. 37934957

[30] P. M. Pöllänen , T. Härkönen , J. Ilonen , et al., “Autoantibodies to N‐Terminally Truncated Gad_65_(96‐585) as a Predictive Marker for Type 1 Diabetes and Hla‐Associations of Gad Epitope‐Specific Autoantibody Responses,” Journal of Clinical Endocrinology and Metabolism 107, no. 3 (2022): e935–e946, 10.1210/clinem/dgab816. 34747488 PMC8851925

[31] J. Ilonen , A. Hammais , A. P. Laine , et al., “Patterns of Β‐Cell Autoantibody Appearance and Genetic Associations During the First Years of Life,” Diabetes 62, no. 10 (2013): 3636–3640, 10.2337/db13-0300. 23835325 PMC3781470

[32] M. Knip , M. Kukko , P. Kulmala , et al., “Humoral Beta‐Cell Autoimmunity in Relation to Hla‐Defined Disease Susceptibility in Preclinical and Clinical Type 1 Diabetes,” American Journal of Medical Genetics 115, no. 1 (2002): 48–54, 10.1002/ajmg.10343. 12116176

