# Supplementary material for: Characteristics of Type 1 Diabetes Among Patients Carrying the Protective HLA‐DQB1*06:02 Allele
Source: HLA. 2024 Nov 20;104(5):e15720. doi: 10.1111/tan.15720 (PMC11586155; doi:10.1111/tan.15720)
Supplement: Supplementary file 1 — Appendix S1. Supporting Information. [file TAN-104-e15720-s001.docx]

Supplementary appendix

Supplement to: Taka A-M, Härkönen T, Vähäsalo P et al. Characteristics of type 1 diabetes among patients carrying the protective HLA DQB1*06:02 allele

**Supplementary material**

The Finnish Pediatric Diabetes Register comprises the following investigators:

***Principal Investigator*:** Mikael Knip (New Children’s Hospital, Helsinki University Hospital)

***Steering Committee:*** Per-Henrik Groop (Folkhälsan Research Center), Jorma Ilonen (Immunogenetics Laboratory, University of Turku), Timo Otonkoski (New Children’s Hospital, Helsinki University Hospital), Riitta Veijola (Department of Pediatrics, Oulu University Hospital).

**Locally responsible investigators:**

Alar Abram (Department of Pediatrics, Kanta-Häme Central Hospital), Henrikka Aito (Department of Pediatrics, HUS Porvoo Hospital), Ivan Arkhipov (Department of Pediatrics, Mehiläinen Länsi-Pohja Central Hospital), Elina Blanco-Sequeiros (Department of Pediatrics, Central Ostrobothnia Central Hospital), Jonas Bondestam (Department of Pediatrics, HUS Lohja Hospital), Markus Granholm (Department of Pediatrics, Jakobstad Hospital), Maarit Haapalehto-Ikonen (Department of Pediatrics, Rauma Hospital), Torsten Horn (Department of Pediatrics, Central Hospital of Central Finland), Hanna Huopio (Department of Pediatrics, Kuopio University Hospital), Joakim Janer (Department of Pediatrics, HUS Raasepori Hospital), Christian Johansson (Department of Pediatrics, Åland Central Hospital), Liisa Kalliokoski (Department of Pediatrics, Kainuu Central Hospital), Päivi Keskinen (Department of Pediatrics, Tampere University Hospital), Anne Kinnala (Department of Pediatrics, Turku University Central Hospital), Maarit Korteniemi (Department of Pediatrics, Central Hospital of Lapland), Hanne Laakkonen (Department of Pediatrics, HUS Hyvinkää Hospital), Jyrki Lähde (Department of Pediatrics, Satakunta Central Hospital), Päivi Miettinen (New Children’s Hospital, Helsinki University Hospital). Päivi Nykänen (Department of Pediatrics, Mikkeli Central Hospital), Erik Popov (Department of Pediatrics, Vaasa Central Hospital), Mari Pulkkinen (Department of Pediatrics, HUS Jorvi Hospital), Maria Salonen (Department of Pediatrics, Kymenlaakso Central Hospital), Pia Salonen (Department of Pediatrics, Päijät-Häme Central Hospital), Juhani Sankala (Department of Pediatrics, Savonlinna Central Hospital), Virpi Sidoroff (Department of Pediatrics, North Karelia Central Hospital), Anne-Maarit Suomi (Department of Pediatrics, South Ostrobothnia Central Hospital, Tuula Tiainen (Department of Pediatrics, South Karelia Central Hospital), Riitta Veijola (Department of Pediatrics, Oulu University Hospital)
